# Active search for COVID-19 cases during integrated supportive supervision using an electronic platform to improve healthcare workers performance in Niger: the legacy of the polio eradication program

**DOI:** 10.11604/pamj.2022.41.187.26820

**Published:** 2022-03-08

**Authors:** Moussa Haladou, Blanche-Philomene Melanga Anya, Batouré Oumarou, Ishagh El Khalef, Joseph Nsiari-muzeyi Biey, Hamidou Harouna, Patrick Katoto, Charles Shey Wiysonge

**Affiliations:** 1 Country Office, World Health Organization, Quartier Plateau, Avenue Mohamed VI 1204, Niamey, Niger,; 2 Epidemics, Surveillance and Response Directorate, Ministry of Public Health, Niamey, Niger,; 3 Sub-Regional Office for West Africa, World Health Organization, Independence Street, Gate 0058, Ouagadougou, Burkina Faso,; 4 Centre for Infectious Diseases, Faculty of Medicine and Health Sciences, Stellenbosch University, Francie van Zijl Drive, Tygerberg 7505, Cape Town, South Africa,; 5 Centre for Tropical Medicine and Global Health, Faculty of Medicine, Catholic University of Bukavu, Bugabo 02, Bukavu, Democratic Republic of Congo,; 6 Cochrane South Africa, South African Medical Research Council, Francie van Zijl Drive, Parow Valley 7501, Cape Town, South Africa,; 7 Department of Global Health, Faculty of Medicine and Health Sciences, Stellenbosch University, Francie van Zijl Drive, Tygerberg 7505, Cape Town, South Africa,; 8 School of Public Health and Family Medicine, University of Cape Town, Anzio Road, Observatory 7935, Cape Town, South Africa

**Keywords:** Poliomyelitis eradication, SARS-CoV-2, surveillance, workforce development

## Abstract

The implementation of electronic data collection during supportive supervision visits (ISS) using the Open Data Kits (ODK) Collection in Niger has provided a factual basis for monitoring the performance of the Polio eradication program (PEP) and the immunization program. With the notification of the first case of COVID-19 on 19 March 2020, there was a rapid need for quality knowledge to monitor the pandemic. For the first time in Niger, we initiated a six-month (May to October 2020) joint ISS-COVID-19 surveillance program to improve and monitor healthcare workers' performance to efficiently investigate COVID-19 cases in eight provinces. Overall, 1,378 ISS visits were performed through 390 health facilities, during which 4,638 health workers were trained and 527,151 medical records were reviewed, of which 28 suspected cases of COVID-19 were found. Field visits for contact tracing in their communities were accomplished and closed monitoring ensured until full recovery. Building on the tradition of PEP, a problem-solving process, feedback and on-the-job training on COVID-19 surveillance is set to enhance notification in the coming weeks and months. This is facilitated by accurate use of ODK Collect for real-time data surveillance successfully implemented. Other topics in the briefing included fundamentals of infection prevention and control for COVID-19 for both health professionals and community leaders. From this experience, the ISS has emerged as a key component of COVID-19 surveillance, especially in regions with a fragile health system. Our observation is a step forward for pragmatic interventional studies.

## Introduction

Developing and deploying a professional workforce to undertake eradication activities has been a core component of the Global Polio Eradication Initiative (GPEI) in Niger. Supportive supervision is one of the steps that fosters program enhancement by imparting health employees with expertise and skills. The fundamental challenge of positive monitoring is the real-time availability of data for timely and efficient input [1]. The integrated supportive supervisory (ISS) checklist is, an android based real-time data collection checklist developed using the Open Data Kit (ODK) tool, used at operational level by supervisors (STOP team, WHO staff, national officers, etc.) [2].

The electronic collection of data from ISS visits using the ODK Collect application provides evidence-based to track operational level activities but, above all, to contribute to the continuous improvement of the performance of vaccination-preventable disease surveillance [3]. The integration of COVID-19 surveillance into this activity is an opportunity to increase its surveillance sensitivity. It allows simple analyses and triangulation to identify better situations that are not easily noticeable. As for Polio eradication program (PEP), substantial material and financial resources are needed to make the best use of new technologies in the health system, to increase the effectiveness of the COVID-19 model for surveillance and for later immunization [2]. The appropriation and optimal use of this tool by the actors on the ground remains a major challenge in monitoring the implementation of interventions for the management of COVID-19 at all levels. We thus analysed the effect of a joint ISS-COVID-19 surveillance program using mobile phones under ODK platform to improve and monitor healthcare workers' performance to efficiently investigate COVID-19 cases in eight provinces in Niger.

## Methods

**Study settings:** this study took place in Niger, a large country in the Sahel area with a rapidly expanding population anticipated to reach 24,811,942 in 2020. As a member of the Central Mediterranean region, it continues to be the world's most perilous irregular migratory route. In Niger, the health sector is divided into three tiers: the central level, which is responsible for developing the general plan and operating national hospitals and health centres; the regional level, which includes the eight *Directions Générales de la Santé Publique*, which are defined by six regional hospitals and two comparison centres; and the third level, which includes 42 Equipes Cadres du District in 42 district hospitals and the associated network of 578 integrated health centres (IHC) and 1201 health areas.

**Figure 1:**
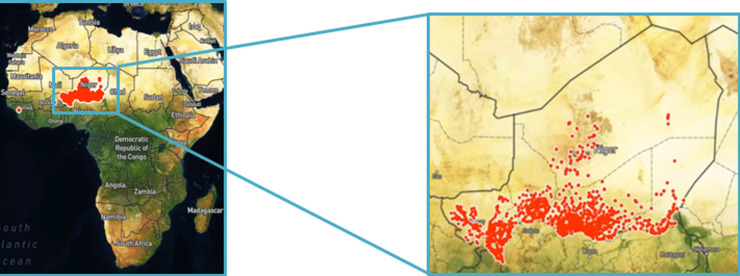
maps of Africa showing Niger. Note: reds dots represent individual data collected via open data collect

**Study design:** a six-month joint ISS-COVID-19 surveillance program (from May 2020 to October 2020) was conducted across all eight political regions of Niger (Niamey, Agadez, Diffa, Dosso, Maradi, Tahaoua, Tillaberi, and Zinder). The program aimed to improve healthcare workers´ performance in efficiently investigating COVID-19 cases. Thus, we reviewed supporting surveillance data obtained during this period through mobile phones by using open data kit (ODK) platform. ODK is a suite of tools that is available for free and is open source. ODK enables users to quickly create a data collecting form or survey, as well as assist the mapping of geocoded data. During the ISS, a GPS coordinate was acquired to determine the precise position of individual data obtained by ODK (Figure 1).

**Data collection and techniques:** after data had been collected at the field level, it was transferred to the central server. The data was then downloaded from the server at the national level in real time. The checklist for routine ISS was used and modified to allow COVID-19 related information to be uploaded to the mobile phones of the supervisors. Supervisors were also trained on basic principle of infectious diseases and control specific to SARS-CoV-2 infection.

**Data analysis:** the central server´s data was exported to a Microsoft Excel spreadsheet (V.14). We quantified the number of weekly/monthly ISS visits by health structures and region, as well as the proportion of such visits that effectively incorporated COVID-19 active case results. Additionally, we utilized a thematic approach to analyse the primary issues raised during ISS, the type of on-the-job training received, and any outcomes attained.

## Results

**Figure 2:**
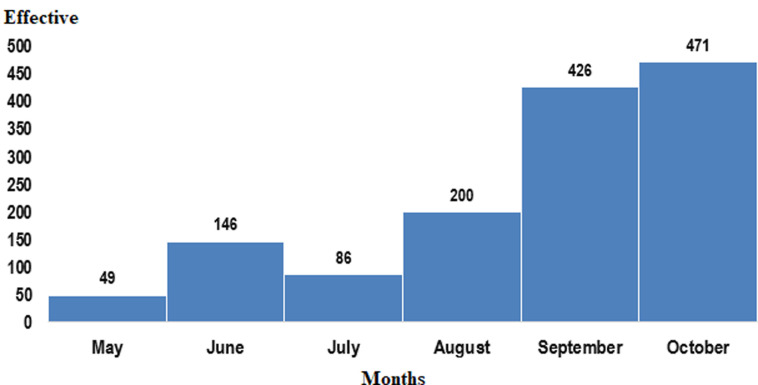
monthly integrated supportive supervisory visits conducted from May to October 2020 in Niger

Between 01 May and 31 October 2020, a total of 1,378 ISS visits were performed in all eight regions, ranging from 80 visits to the Niamey region to 336 visits to the Zinder region. The regions of Zinder, Diffa and Tahoua were responsible for 24 percent, 20 percent, and 14 percent of all visits respectively (Table 1). During these supervisions, 999 visits (72.5%) integrated the active search for COVID-19 cases, ranging from 56.7% in Tahoua to 97.7% in Tillabery. Increased ISS production was observed between epidemiological week 35 and week 41 of 2020. A decrease in the number of visits was observed during the month of July, a decrease justified by the lack of funding for field visits by the Polio Eradication Program. A progressive increase in visits is observed in August, September, and October with 200, 426 and 471 visits, respectively (Figure 2).

**Table 1:** problems identification, supportive measures and results obtained to strengthen active COVID-19 case findings at health care facilities level

Region	ISS Visits without COVID-19 Surveillance N (%)	ISS Visits with COVID-19 Surveillance N (%)	Total ISS Visits N (%)
Zinder	101 (30.1)	235 (69.9)	336 (24.4)
Tahoua	119 (43.3)	156 (56.7)	275 (20.0)
Diffa	81 (41.5)	114 (58.5)	195 (14.2)
Maradi	12 (6.45)	174 (93.5)	186 (13.5)
Tillaberi	3 (2.5)	117 (97.5)	120 (8.7)
Agadez	13 (12.9)	88 (87.1)	101 (7.3)
Dosso	34 (40.0)	51 (60.0)	85 (6.2)
Niamey	16 (20.0)	64 (80.0)	80 (5.8)
Total	379 (27.5)	999 (72.50)	1,378 (100)

ISS: integrated supportive supervisory

This increase is explained by the deployment of new consultants and the financing of monthly field trips. However, there was a decline in visits at week 44 in all due to the organization of the response to circulating poliovirus type 2 derived from vaccine. The overall number of visits conducted depended on the availability of resources (human and financial) and the willingness to use the ODK tool to ensure scheduled visits. For example, the Diffa, Tahoua and Zinder region with different consultants, such as Auto-visual acute flaccid paralysis (AFP) detect and report (AVADAR) consultants, Stop Team and WHO staffs (National Professional Officer) benefited from more visits compared to those with fewer consultants, such as the Niamey Region, assisted by only two Consultants. Despite, being in unsecured situation and given the restricted movement imposed by the government, Tillabery region has good performances mainly due to fact that most of the visits were conducted in cities. In most cases, inadequate preparation of visits has been linked to a decline in the number of visits across all regions. In particular, the financial issue should be addressed earlier to ensure smooth ISS visits. In our cases, a question of financial resources has been established for the continuation of joint supervision with the government.

**Figure 3:**
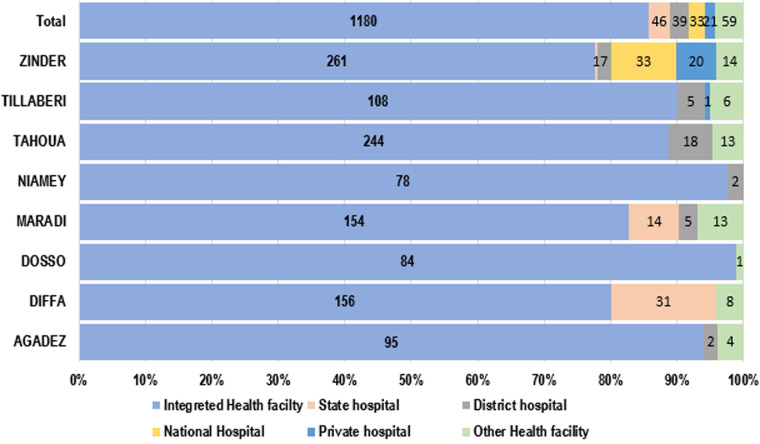
display of conducted integrated supportive supervisory visits by type of health structures visited (n=1378)

**Figure 4:**
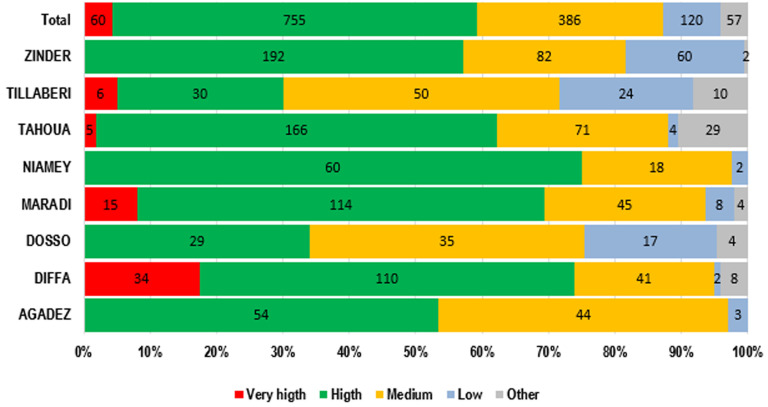
integrated supportive supervisory visits carried out according to the type of health structures attendance (n=1378)

Considering the type of health structure that was visited during the ISS, 85.63% of all visits (1378), were conducted in the integrated health centers (IHC). In the Dosso and Zinder areas, the bulk of ISS visits were conducted in this group of health facilities. However, 8.56% visits only have been made to district, regional and national hospitals, which are the main structures of the health system pyramid in Niger (Figure 3). Overall, active searches were performed for reported cases of COVID-19 in 370 IHC, 5 private health facilities, 10 district hospitals and 5 regional hospitals. In the light of this study, it is important to carry out this operation at the level of hospitals and major health centres with high attendance (high priority) and accessible for scheduled visits to maximize the anticipated outcomes (Figure 4).

A total of 4638 health workers received on-the-job training for COVID-19 surveillance during the 1,378 ISS visits conducted to directly enhance daily notification. Overall, an average of 3.4 health workers received briefings on COVID-19, with a variation ranging from 15 health workers in Niamey to 7.4 in the Diffa region. An analysis of the consultation registers reported 527,151 patients at the level of the healthcare facilities visited. During the five months, 28 suspected cases of COVID-19 were identified in the country, of which five were investigated and detected in the regions of Diffa (2 cases), Zinder (2 cases) and Niamey (1 case). As a result, the ISS visits provided for a rapid investigation of these cases and for community-level contact tracing. This rapid and controlled workout has allowed better management and reintegration of these patients into the community at discharge. The most often encountered challenges (Table 2) during the ISS may be classified into five axes, with most centres reporting concerns with resource management and training.

**Table 2:** integrated supportive supervisory visits across eight regions in Niger from May to June 2020 (n=190): problem’s identification, supportive measures and results obtained to strengthen active COVID-19 case findings at health care facilities level

Region	Major Problems Identified	On-the-job training/Corrective Actions	Results
Agadez	* Poor knowledge of COVID-19 surveillance procedures by HCF managers	* Intensification of remote area supervision missions to reach HCF managers	Appropriation of COVID 19 surveillance by health workers with case notification
	* Low GSM coverage: no access to updates on COVID-19	* Refreshing case definitions, roles, and responsibility of HW in COVID-19 surveillance	
Diffa	*Dysfunction of the ODK tool	*Interaction with the national level and WHO Africa for a downable form	*Installing the tools and carrying out visits using the ODK Tool
	*Inadequate funding for ISS exits	*Submission of Reference Terms for 5-day outings in July 2020.	
			*Conducting 52 visits
	*Inadequate staff training (case definition)	*Officers' awareness of COVID-19 cases definition and procedures for reporting and investigating suspected cases	
Dosso	* Logistic issue with STOP TEAM	* Problem raised during the meeting with WHO representative	*Problem solved
	* Delay in setting up funds for the implementation of community-based surveillance	* Request sent 2 weeks ago	*Request pending
			*Decrease in active surveillance
		*Funds sent systematically by the central level	
	* Delay in setting up funds to health districts for integrated active surveillance of AFP		
Niamey	* Decrease in patient attendance at HCF	* Raising community awareness by CHW	*Gradual resumption of patients’ attendance
			*District managing teams trained on COVID-19 monitoring
	* Not systematic use of COVID-19 case definition by health care providers	* Training/refreshing on the use of case definitions	
			*Active search with ODK started in Niamey District 5, pilot district
		*Practical work on COVID-19 surveillance using ODK Collect Tool	
Maradi	*Low number of HW trained on COVID-19 surveillance	*Training when visiting priority sites	*Appropriation of active COVID 19 surveillances by HW.
			*Improvement of surveillance indicators
		*Drafting case definition posters	
	* Hard to train 5 HCF around conflict zones. (Border with Nigeria)		
Tahoua	* Logistical issues (cars, motorbikes) since early June 2020	* Temporary use of other district service vehicles during integrated supervision	* Implementation of surveillance activities in some structures in the region.
	* Lack of human resources (one staff)	* Information sent to the hierarchy (WHO) for the resolution of the logistical problem	* Commitment by stakeholders to improve the use of ODK
	* Delay in making outflow funds available		
	* Inadequate use of ODK tool by district actors when visiting sites	* Local actors recalled for effective use of ODK by the regional directorate of public health	
		* Field visits with the head of the Health Programming and Information Service and the heads of the Epidemiological Surveillance Centers of visited districts	
Tillabéri Municipality	* Non-systematic wearing of mask during patients visit	*Refresh on IPC (modes of transmission, highlight of droplets kinetic during sneezing or coughing).	HWs wear mask and appear to have clearly perceived it usefulness
	* Inadequate systematic awareness on COVID-19 of people attending HCF	* A reminder of the importance of communication, mainly regarding preventive measures (social distancing, wearing mask, hand washing, opening windows)	*Commitment by HW to raise awareness
Zinder	* Unsatisfactory effort by government	*Update of the situation to the national surveillance supervisors	Request pending
	* Inadequate funding from the heads of the sub-office (national professional officer)	*Option to use emergency funds	

Abbreviations: HCF: health care facility; HWs: health workers; CHW: community health worker; WHO: world health organisation; AFP: acute flaccid paralysis: STOP TEAM: The Stop Transmission of Polio (STOP) program; ODK: open Data Kit.

## Discussion

Our study analysed the effect and challenges of a joint ISS-COVID-19 surveillance program using mobile phones under ODK platform to improve and monitor healthcare workers' performance to efficiently investigate COVID-19 cases in eight provinces in Niger. Overall, 1,378 ISS visits were performed through 390 health facilities, during which 4,638 healthcare workers were trained and 527,151 medical records were reviewed, of which 28 suspected cases of COVID-19 were found and 10 cases confirmed. Field visits for contact tracing in their communities were accomplished and closed monitoring was ensured until full recovery. Strikingly, on-the-job training for COVID-19 surveillance was conducted as well as skill transfer ensured.

A study in Pakistan found that improving supervisors “supervisory skills enhances community health workers” ability to diagnose and handle infectious diseases compared to conventional supervision if it is essential to help them through refresher training, logistics and commodities [4]. However, several factors may impact the activities of health professionals, some beyond the direct control of program managers. An awareness of these factors will help inform the development of performance improvement strategies [5]. In this humanitarian setting, our study has enumerated numerous factors that will contribute to strengthen health system and support effective measure for COVID-19 surveillance and management. Firstly, the issue of COVID-19 surveillance among health care workers (HCWs). This ranges from inadequate general knowledge (Agadez) to incorrect use of the case description of COVID-19 (Niamey and Diffa) and to inappropriate application of fundamentals principles of infection prevention and control (IPC) for COVID-19 such as wearing a face mask during patient visit (Tillabéri Municipality). To this end, the ISS team set up to refresh HCWs with the IPC principles for COVID-19. In order to achieve this aim, the ISS team conducted remote supervision for HCW members, increased training frequency with focus on the four Ws (washing hand, wearing face mask, watching distance and windows held open).

Secondly, problems related to human resources. This ranges from a low proportion of qualified HCWs and hard-to-reach HCWs across conflict areas (Maradi) to a complete lack of human capital (Tahoua). To enhance monitoring in such an environment, further preparation for staff training has been planned remotely, and a deeper debate has taken place at the district level to ensure new recruitment where needed. Thirdly, issues related to material capital. This ranges from a lack/ insufficient of funds to achieve a success ISS (Diffa) to a delay in setting up funds for community-based surveillance and for the district to combine active surveillance of acute flaccid paralysis (Dosso and Zinder) as well as to logistical difficulties such as the availability of transport means (Tahoua). Greater discussions took place at various levels for earlier funding forecasting and for engineering intermediate options such as using cars for other services at district level while waiting for funds to be systematically sent from the central level. Fourthly, the reasonably low rate of use of ODK and the electronic platform to boost surveillance of COVID-19. This ranges from poor GSM coverage (Agadez) to ODK instability (Diffa) to insufficient use of ODK by district actors (Tahoua). The ISS team then concentrated on installing software, conducting visits using ODK with real world data to promote successful case searching for COVID-19. Moreover, stakeholders were also engaged in various campaign to enhance the use of ODK. Lastly, patients related problems. This varies from pandemic induced hysteria that leads to a decrease in patient attendance at health facility level (Niamey) to the inadequate commitment to use IPC related COVID-19 interventions such as systematic handwashing and wearing of face mask. Gradual resumption of patients´ attendance and adherence to IPC principles were observed after both HCWs and community leaders increased community consciousness.

Solutions to these issues may contribute to the overall improvement of the health system, as well as disease surveillance. In Kenya, the STOP program, a network of trained public health practitioners who supported with training and skill transfer, ensured the ISS's sustainability and helped in the reporting of missing AFP cases at health facilities and in the community [3]. Similarly, by combining polio eradication program (PEP) supervision with COVID-19 monitoring, we were able to identify and effectively trace 10 missing cases of suspected COVID-19 at the community level. As a result of ISS's contribution to an increase in non-polio AFP detection rates, the approach has boosted the sensitivity of AFP surveillance. As such, it is reasonable to predict that intensive search at the community level in conjunction with good contact tracing will aid in containing the pandemic in the community for COVID-19. Additionally, the low-cost technology underlying ISS's use of ODK will facilitate the implementation of planned supervisions, allowing for the transfer of expertise and skills and encouraging local capacity building, all of which will strengthen the fundamentals of COVID-19 surveillance, just as it did for AFP [1, 2, 6].

## Conclusion

During ISS visits, thousands of health professionals got on-the-job training in COVID-19 surveillance witch directly enhanced routine notification in Niger. Supportive management has prompted employees to continually improve their own job performance. This was beneficial during the early months of the pandemic, as it supported healthcare professionals in a supportive and non-authoritarian manner in time of high professional stress and on developing team methods that promote problem-solving. Keeping in mind the dynamic epidemiologic curve (several waves) of the COVID-19 pandemic, adopting portable devices with ODK for case surveillance will give real-time data and feedback from health facilities to state/zonal levels. Additionally, when combined with ISS, it can assist in quickly correcting process indicators and facilitating skill transfer during a pandemic.

### What is known about this topic

- In polio-affected countries, suboptimal programmatic output is related to a lack of qualified human resources, and it is described as a major challenge to polio eradication;
- Integrated supportive supervision (ISS) promotes improved communication, teamwork to address problems, and mentorship to drive healthcare workers to supervise, monitor, and improve their individual and collective performance;
- Open Data Kits (ODK) using mobile data collection has been associated with improving expended programme on immunization (EPI) activities, monitoring, and performance in various low- and middle-income countries.

### What this study adds

- The implementation of electronic data collection during ISS using ODK in Niger has provided a factual basis for monitoring the performance of COVID-19 surveillance by leverage on EPI activities;
- Building on the tradition of PEP, a problem-solving process, feedback and on-the-job training on COVID-19 surveillance was possible during ISS;
- ISS emerged as a key component of COVID-19 surveillance and health system strengthening, especially in regions with a fragile health system.
